# Descriptive Histopathological and Ultrastructural Study of Hepatocellular Alterations Induced by Aflatoxin B1 in Rats

**DOI:** 10.3390/ani11020509

**Published:** 2021-02-16

**Authors:** Fatma Abo Zakaib Ali, Fatma M. Abdel-Maksoud, Hekmat Osman Abd Elaziz, Ashraf Al-Brakati, Ehab Kotb Elmahallawy

**Affiliations:** 1 Department of Pathology and Clinical Pathology, Faculty of Veterinary Medicine, Sohag University, Sohag 82524, Egypt; fatma_ali@vet.sohag.edu.eg; 2 Department of Anatomy and Histology, Faculty of Veterinary Medicine, Assiut University, Assiut 71526, Egypt; fatma.abdelmaksoud@vet.au.edu.eg; 3 Department of Histology, Faculty of Medicine, Sohag University, Sohag 82524, Egypt; hekmatosman@yahoo.com; 4 Department of Human Anatomy, College of Medicine, Taif University, P.O. Box 11099, Taif 21944, Saudi Arabia; a.albrakati@tu.edu.sa; 5 Department of Zoonoses, Faculty of Veterinary Medicine, Sohag University, Sohag 82524, Egypt

**Keywords:** aflatoxin B1, fibrosis, Ito cells, Kupffer cells, necrosis, ultrastructure

## Abstract

**Simple Summary:**

Aflatoxins can affect hepatocytes, which results in a series of histological and ultrastructural changes to the cells. We investigated the hepatocellular alterations induced by aflatoxin B1 in rats. Interestingly, we observed several histopathological and ultrastructural alterations in hepatocytes, including necrotic changes and massive vacuolar degeneration. Ultrastructural examinations of treated groups revealed damage to the sinusoidal endothelium, as well as aggregations of hyperactive Kupffer cells in the space of Disse and damaged telocytes. Our findings provide novel insights into the induction of a series of irreversible adverse effects on hepatocytes by aflatoxin B1. Based on our results, we suggest future investigations for the exploration of mechanistic pathways related to these induced hepatocellular alterations.

**Abstract:**

Liver sinusoids are lined by fenestrated endothelial cells surrounded by perisinusoidal cells, Kupffer cells, and pit cells, as well as large granular lymphocytes. The functional ability of the liver cells can be substantially modified by exposure to toxins. In the current work, we assessed the histopathological and ultrastructural effects of a time-course exposure to aflatoxin B1 (AFB1) on the hepatic structures of rats. A total of 30 adult female Wistar rats were randomly divided into three groups: a control group, a group orally administered 250 µg/kg body weight/day of AFB1 for 5 days/week over 4 weeks, and a group that received the same AFB1 treatment but over 8 weeks. Histopathological and ultrastructural examinations of hepatocytes revealed massive vacuolar degeneration and signs of necrosis. Furthermore, the rat liver of the treated group exhibited damage to the sinusoidal endothelium, invasion of the space of Disse with hyperactive Kupffer cells, and some immune cells, as well as Ito cells overloaded with lipids. In addition, damaged telocytes were observed. Taken together, our results indicate that AFB1 induces irreversible adverse effects on the livers of rats.

## 1. Introduction

Mycotoxins are secondary metabolites of toxigenic fungi. Aflatoxins are a family of mycotoxins produced by *Aspergillus* spp. [1,2]. According to the Food and Agriculture Organization, about a quarter of the crops in the world are affected by mycotoxins [3,4]. These health-harming toxins have been detected as pollutants during various agronomic processes in several regions that have warm and moist weather [2,5,6]. Aflatoxins are extremely toxic and can cause serious pollution to dietary sources. Worryingly, contamination by aflatoxins has been reported in grains during preharvest and postharvest conditions [7]. The ingestion of aflatoxin-contaminated foodstuffs can lead to severe health problems in humans and animals [8,9,10]. Consequently, the safe dose limit for human consumption of aflatoxins is only 4–30 µg/kg [11]. During the metabolism of aflatoxin B1 (AFB1), cytochrome P450 is stimulated, and reactive oxygen species (ROS), such as superoxide ions and hydrogen peroxide, are produced, which can lead to marked oxidative stress that must be counteracted by antioxidants [12,13]. However, where oxidative stress occurs at levels greater than the protective capacity of antioxidants, critical macromolecules, including lipids, DNA, and proteins, may be damaged. In addition, biological functions, such as calcium influx, membrane leakage, and DNA stability, may be affected, which can ultimately lead to cancer [13,14,15]. Furthermore, high levels of ROS can destroy liver hepatocytes [16]. In addition, aflatoxins are mutagenic and induce carcinogenic effects in the liver and other organs [17]. However, while several previous studies have largely focused on the pathological effects of AFB1 on liver tissue, the effects of AFB1 on the fine structure of hepatocytes and other cells, such as Kupffer, Ito (perisinusoidal), and pit cells, remain unclear. Thus, in the current study, we aimed to determine the influence of AFB1 on the fine structure of the hepatic system, and to elucidate the histopathological changes associated with experimental aflatoxicosis.

## 2. Materials and Methods

### 2.1. Chemicals

AFB1 (Sigma-A6636), a white to light yellow odorless powder, was dissolved in olive oil (used as a vehicle).

### 2.2. Animals and Experimental Design

All experimental and euthanasia procedures were performed in accordance with a protocol approved by the research ethics committee of the Faculty of Veterinary Medicine, Sohag University, Egypt. All procedures used in this study were approved by the Research Ethics Committee of the Faculty of Veterinary Medicine, Sohag University, Egypt. A total of 30 adult female Wister rats weighing 150–250 g were allowed to acclimatize for 7 days at the Faculty of Veterinary Medicine, Division of Laboratory Animal Health Housing Facility. The animals were maintained in a 12:12 h light/dark cycle and an ambient temperature of 20–23 °C; they were provided with food and water ad libitum. After the acclimation period, the rats were randomly divided into three groups. Group I, the control group, was further subdivided into two subgroups, each consisting of 10 rats, 5 of which were sacrificed after 4 weeks. The remaining five were sacrificed after 8 weeks (consistent with groups II and III below, respectively). In subgroup IA, the animals (*n* = 10) were provided with water ad libitum, fed a standard diet, and maintained without any treatment. In subgroup IB, the rats (*n* = 10) received the olive oil vehicle (0.2 mL/animal/day) orally through a gastric tube. In group II, the rats (*n* = 5) were orally administered 250 µg/kg body weight/day of AFB1 [18,19], which was dissolved in olive oil as a vehicle [19,20], through a gastric tube 5 days/week for 4 weeks. In group III, the rats (*n* = 5) received the same AFB1 treatment as in group II but for 8 weeks [18,21,22].

### 2.3. Specimen Processing and Staining

At the end of the respective experimental periods, liver specimens were obtained after whole-body perfusion of experimental rats with 4% paraformaldehyde (catalog no. 19200; lot no. 090820; Electron Microscopy Sciences (JEOL, Tokyo, Japan)). The samples were dissected and immediately fixed in 10% formalin for 24 h, after which they were dehydrated in a graded alcohol series, cleared in xylene, and finally embedded in paraffin. The tissue was cut into 3 μm thick sections and then stained with hematoxylin and eosin [23]. Histopathological observations were performed using an Olympus CX 41 RF light microscope (Olympus Corporation, Tokyo, Japan).

### 2.4. Ordinal Method for Validating Histopathologic Scoring

Each animal was assigned a score based on tissue histopathological examination [24]. The samples were scored quantitatively and semiquantitatively, with assessment based on the visual field inspection of a minimum of 10 sections from each group. Photographs were taken at a magnification of 40×, and the cell numbers of hepatocyte alterations (vacuolar degeneration, binucleated hepatocytes, and megalocytes) were counted in 10 randomized areas (each 1 mm^2^) [16].

In addition, the severity of periportal fibrosis was scored as follows: 0 = no lesions; 1 = minimal (1–10% of the tissue section affected); 2 = mild (11–25%); 3 = moderate (26–45%) and 4 = severe (>45%) [24].

### 2.5. Semi-Thin Section Preparation and Transmission Electron Microscopy

Small liver specimens were fixed in 2.5% paraformaldehyde and glutaraldehyde in 0.1 M Na–cacodylate buffer (pH 7.2) for 24 h at 4 °C [24]. These samples were then washed in the same buffer before being postfixed in 1% osmic acid in 0.1 M Na–acodylate buffer for 2 h at room temperature. Subsequently, the samples were dehydrated in ascending grades of ethanol and embedded in an Araldite–Epon mixture. Semi-thin sections were cut at a thickness of 1 µm before being stained with 1% Toluidine blue; Suvarana et al. [25] described all the staining methods from Bancroft’s theory as well as the histological techniques. The stained sections were first examined using a Leitz Dialux 20 microscope 35578 Wetzlar, Germany, and photographs were taken using a Canon digital camera (Canon PowerShot A95, China). For transmission electron microscopy (TEM), ultrathin sections were stained with uranyl acetate and lead citrate and then photographed under a JEOL 100 II transmission electron microscope (JEOL, Tokyo, Japan) at the Electron Microscopy Unit of Assiut University.

### 2.6. Digital Colorization of TEM Images

To increase the visual contrast between several structures on the same electron micrograph, we digitally colored specific elements to increase their visibility. All elements of interest were carefully hand-colored using Adobe Photoshop (version 6).

### 2.7. Statistical Analysis

Data were expressed as means ± standard deviations. Data from experimental groups were statistically analyzed using one-way ANOVA with Tukey’s post hoc multiple comparisons tests using the GraphPad Prism software version 5 (San Diego, CA, USA). *p* < 0.05 was used to define statistically significant differences between the groups [26].

## 3. Results

**Figure 1:**
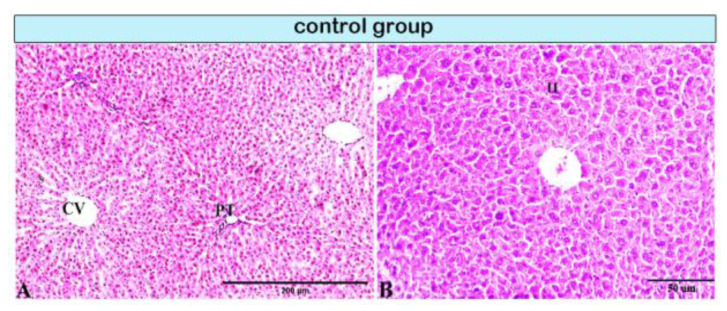
Photomicrograph of a rat liver in the control group demonstrating normal hepatic architectures. (**A**) Central vein (CV), sinusoids (S), and intact portal tirade (PT). (**B**) Hepatocytes (H).

Pathological changes in the liver tissue were observed in all experimental groups except for the control group, which exhibited an intact hepatic architecture in hepatic lobules with normal portal areas (Figure 1A,B, and Figure 4A). Histological changes were not observed in the hepatic tissue of rats from control subgroup IB when compared with that of control subgroup IA across experimental durations.

**Figure 2:**
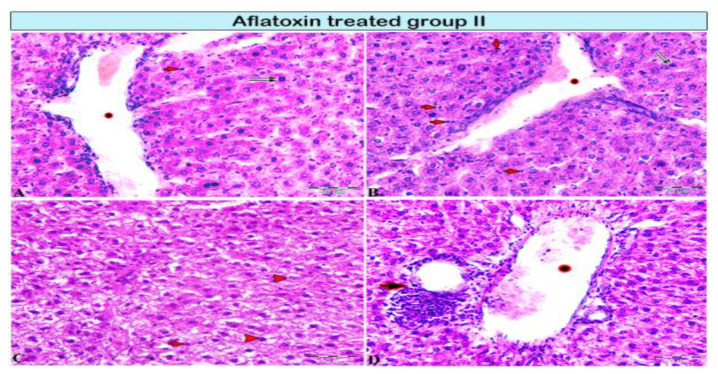
Photomicrograph of a rat liver in aflatoxin B1-treated group II (4-week treatment). (**A,B**) Central vein dilation (star), mononuclear cell infiltration (red arrows), cellular necrosis (pyknotic nucleus; double black arrow), incomplete mitotic division (A: black arrow). (**C**) Vacuolar degeneration of hepatocytes (red arrowheads). (**D**) Interlobular vein extensively dilated and congested with blood (star), periportal interlobular bile duct fibrosis with focal mononuclear cellular infiltration (arrow).

**Figure 3:**
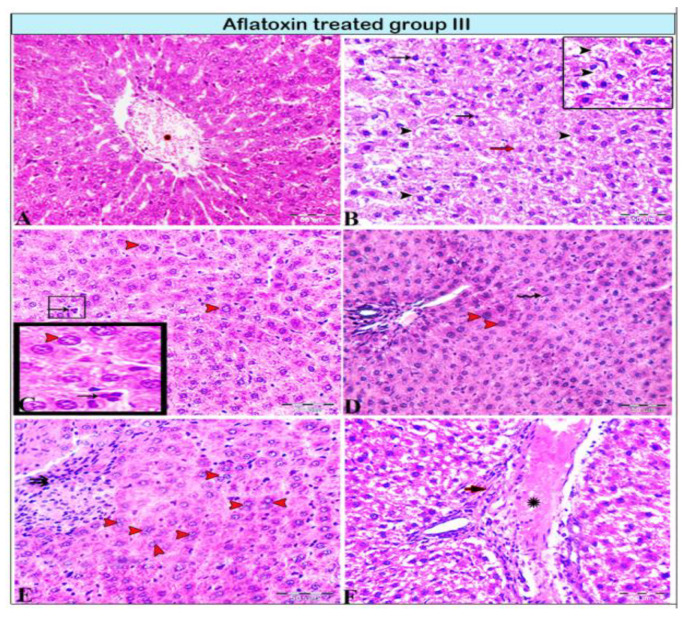
Photomicrograph of a rat liver in aflatoxin B1-treated group III (8-week treatment). (**A**) Extensive central vein congestion and thrombosis (star). (**B**) Vacuolar degeneration of hepatocytes (black arrowheads, magnified in the black square) and hepatocellular necrosis; pyknotic cellular nucleus (black arrows) or karyolitic nucleus (red arrow). (**C,D**) Hepatic megalocytes (red arrowheads), abnormalities in mitosis with tripolar mitosis (C: squares and black arrows, respectively), and Kupffer cell proliferation (D: black arrows). (**E**) Degenerated binucleated hepatocytes (red arrowheads); a focal area of necrosis; several adjacent hepatocytes are absent and replaced by inflammatory cells (double arrows). (**F**) Marked dilatation in the portal vein (star) with periportal fibrosis (arrow).

**Figure 4:**
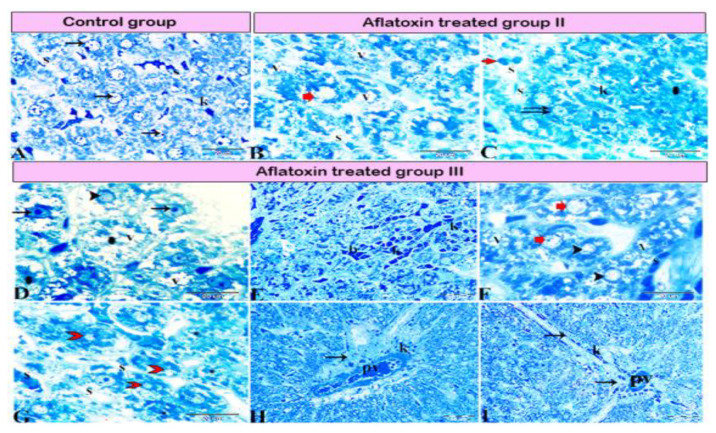
Photomicrograph of a semi-thin section stained with Toluidine blue. (**A**) Normal hepatocytes (arrows) with narrow blood sinusoids (S) in between; note the presence of Kupffer cells (K) in the sinusoids. (**B,C**) Aflatoxin B1-treated rat from group II. (**B**) hepatocellular vacuolar degeneration (V), dilated sinusoids (S), and presence of hepatic megalocytes (red arrow). (**C**) Binucleated hepatocytes (double arrow), incomplete mitotic division (red arrow), area of necrosis (star), dilated sinusoids (S), and marked Kupffer cell (K) proliferation inside the lumen. (**D**–**I**) AFB1-treated rat from group III. (**D**) Severe hepatocellular vacuolar degeneration (V), some hepatocytes with basophilic bodies (arrows), vesicular nuclei (arrowhead), and other hepatocytes showing necrosis with karyolitic nuclei (stars). (**E**) Sinusoids dilated and filled with blood (b) and marked Kupffer cell (K) proliferation inside the sinusoidal lumen. (**F**) Hepatocellular vacuolar degeneration (V), presence of hepatic megalocytes (red arrows), and necrotic hepatocytes (arrowheads). (**G**) Hepatocellular necrosis (red arrowhead) with notable areas of cellular necrosis and lost detail (star). (**H**) Severe periportal fibrosis (arrow), with the portal vein (PV) engorged with blood, severe fibrosis, and marked Kupffer cell proliferation (K). (**I**) Marked fibrosis around the portal vein (PV), which diffused inside the hepatic lobule forming tracts of fibrosis (arrows).

**Figure 5:**
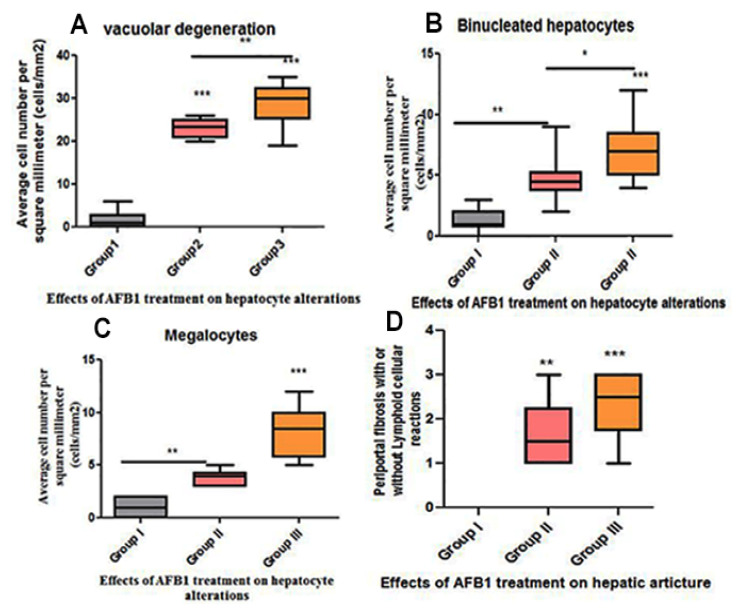
Histomorphometry graph showing semiquantitative measurements of hepatocyte changes among experimental groups. (**A**) Vacuolar degeneration; (**B**) binucleated hepatocytes; (**C**) average megalocytes (cells/mm^2^) among the groups; and (**D**) periportal fibrosis score with or without periportal lymphocytic cellular reaction. Data are expressed as means ± standard deviations. Significant differences vs. the control group are marked by different asterisks through one-way ANOVA with Tukey’s post hoc test: * *p* ≤ 0.05, ** *p* ≤ 0.01, *** *p* ≤ 0.001).

Livers from group II rats (4-week AFB1 treatment) demonstrated central vein dilatation and congestion (Figure 2A,B) and enormous hepatic vacuolar degeneration across the entirety of the hepatic lobules (Figure 2C and Figure 4B). Some cells exhibited mitotic abnormalities in the form of tripolar mitosis (Figure 2B). Focal hepatocellular necrosis and Kupffer cell proliferation were observed (Figure 4C). The interlobular vein was distended and congested with blood. Furthermore, interlobular bile duct hyperplasia with periportal fibrosis was observed (Figure 2D). The rats in group III (8-week AFB1 treatment) exhibited severe vein congestion and thrombosis (Figure 3A), as well as enormous hepatic vacuolar degeneration (Figure 3B and Figure 4D). The diameter of some hepatocytes was less than that of the other neighboring cells, and hypereosinophilic cytoplasm was observed due to the accumulation of pyknotic nuclei within the cytoplasm (Figure 3B and Figure 4F,G). Some hepatocytes demonstrated pale or absent nuclei (Figure 3B and Figure 4D,F,G). Notably, the rats from group III exhibited megalocytes (hypertrophic hepatocytes) (Figure 3C,D and Figure 4F). These hypertrophic cells cause hepatic cord disruption. Several binucleated hepatic cells were observed (Figure 3E), as were some cells exhibiting a high rate of mitotic abnormalities in the form of tripolar mitosis (Figure 3C). Spotty focal areas of necrosis, minute clusters of hepatocytes, the absence of adjacent hepatocytes/their replacement with lymphocytes, and proliferated Kupffer cells were all observed (Figure 3E). Moreover, massive periportal fibrosis, bile duct hyperplasia, and excessive portal vein congestion with inflammatory cell infiltration were noted in all portal areas (Figure 3F, Figure 4H and Figure 5D). In some areas, fibrosis was observed around the portal veins, which extended as tracts inside the hepatic lobules (Figure 4I and Figure 5D). A significantly high number of megalocytes, binucleated hepatocytes, and different patterns of mitotic abnormalities were apparent in group III compared with the indicated malignancies in the other groups. Vacuolar degeneration in group III was significantly higher than that in group II (*p* < 0.05), both relative to the control group (Figure 5A). The number of binucleated cells in group III was significantly greater than that in group II (*p* < 0.05; Figure 5B), both relative to the control group. Finally, the number of megalocytes was significantly higher in group III than in group II (*p* < 0.05; Figure 5C).

### Transmission Electron Microscopy

**Figure 6:**
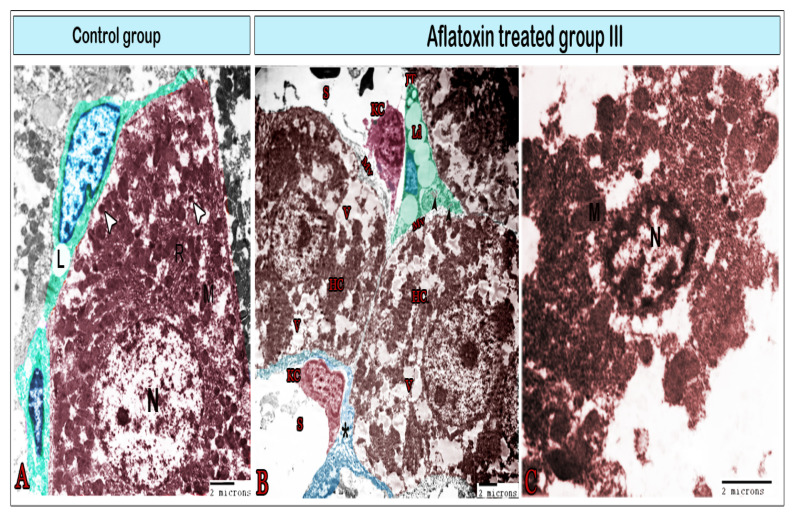
Digital colored transmission electron microscopy micrographs of the control group (**A**) and aflatoxin B1-treated group III (**B,C**). (**A**) Normal hepatocytes with a large round nucleus (N); cytoplasm contains the mitochondria (M), rER, and lysosomes (arrowheads); Ito cells (green color) surrounding the hepatocyte contain lipid droplets (L). (**B**) Hepatocytes (HC) demonstrated exaggerated amounts of vacuoles (V); microvilli (MV) on their surface; Ito cells (IT) with a significant increase in lipid droplets (Lp) that condense the nucleus (blue), and collagen bundles within the cell (arrowheads). Kupffer cells (KC) were observed within the sinusoids (s), which were surrounded by a thick layer of fibrous tissue (*). (**C**) Necrotic hepatocyte exhibiting vacuolation, karyolysis, plasma membrane rupture, and release of cellular contents (note the nucleus (N) and mitochondria (M)).

**Figure 7:**
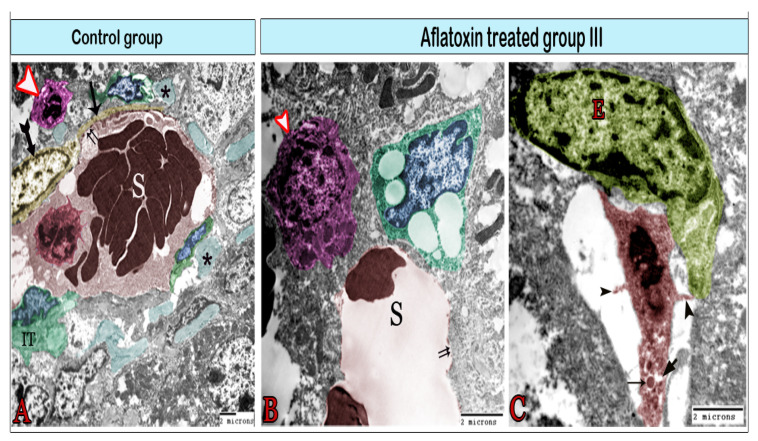
Digital colored transmission electron microscopy micrographs of the control group (**A**) and aflatoxin B1-treated group III (**B,C**). (**A**) Blood sinusoid (S) lined with fenestrated endothelium (double arrows). The lumen of the sinusoid contains Kupffer cells (red). The blood sinusoid is surrounded by telocytes (TCs; yellow), Ito cells (IT) containing fat droplets, and pit cells (arrowhead). Note the TCs’ cell body (biforked arrow), telopodes (arrow), and bundles of collagen fibers (*). (**B**) Blood sinusoid in the aflatoxin B1-treated group exhibiting a large gap in the endothelial lining (double arrows). It is surrounded by enlarged pit cells (arrowhead) and Ito cells (IT) which are overloaded with large fat droplets. (**C**) Kupffer cells (red) are located within the sinusoid and attached to the endothelial cell (E) through its cytoplasmic extensions (arrowheads). They contain lysosomes (arrow) and vacuoles (V).

**Figure 8:**
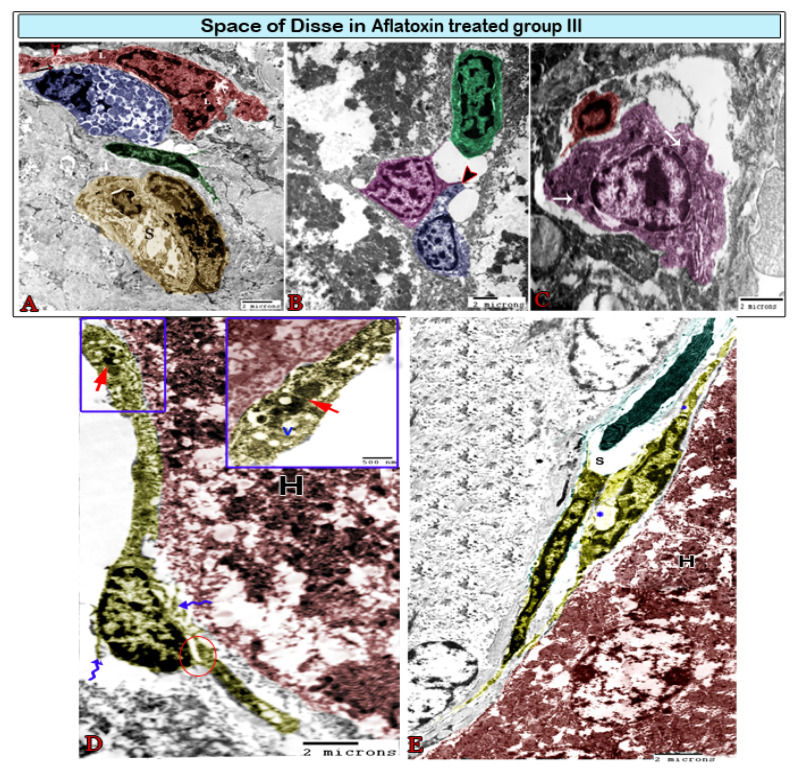
Digital colored transmission electron microscopy micrographs of the space of Disse in aflatoxin B1-treated group III. (**A**) Hyperactive large Kupffer cells (red) sending long processes and containing numerous heterogeneous lysosomes (L) and phagosomes (arrowhead). They are in contact (arrow) with mast cells (blue), which are easily recognizable by their granulations; note the blood sinusoids (S) surrounded by pericytes (green), which are considered to be modified perisinusoidal cells. (**B**) Plasma cells (green) in association with dendritic cells (magenta), which send out their processes (arrowhead) and come into contact with lymphocytes (blue). (**C**) Pit cells (magenta) in contact with Kupffer cells (red). They are recognizable by their dense granules (arrow). (**D**) The telocytes (TCs; yellow) surrounding the hepatocyte (H) had cell bodies encircled by dissolute plasma membranes (wavy arrows) and partially dissociated TPs (circle). TPs contain cytoplasmic vacuoles (v) and heterogeneous lysosomes (red arrow). (**E**) TCs (yellow) exhibiting direct homocellular contact with other TCs. They were characterized by the small perinuclear cytoplasm located between the blood sinusoid (S) and hepatocyte (H), and their cytoplasm contained vacuoles (*).

**Figure 9:**
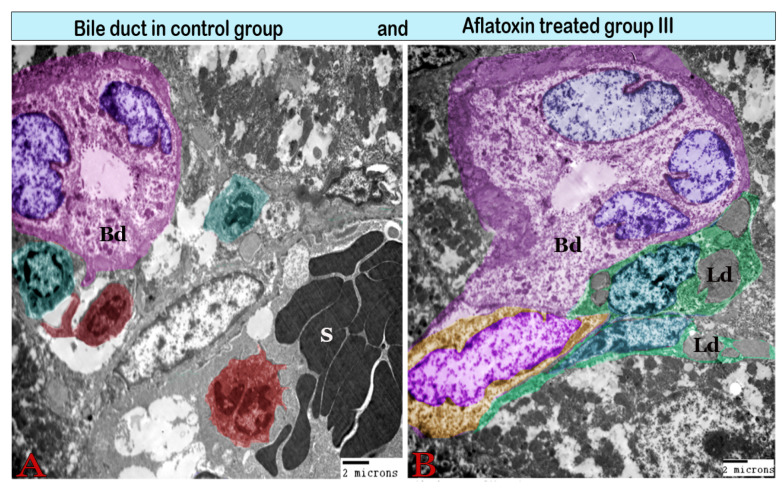
Digital colored transmission electron microscopy micrographs of a cross section of a bile duct (Bd) from the control group (A) and aflatoxin B1-treated group III (B). (**A**) Normal interlobular bile duct surrounded by Kupffer cells (red) and pit cells (green); note the lumen (L) and bile duct epithelium (E). (**B**) Large interlobular bile duct encircled by a thick fibrous sheath. It is surrounded by Ito cells (green), which are loaded with large lipid droplets (Ld).

TEM was employed to further investigate the effects of AFB1 administration for 8 weeks on hepatic cells and sinusoids (Figure 6, Figure 7, Figure 8 and Figure 9). In the control tissue, hepatocytes exhibited a quadrilateral shape with a rounded euchromatic nucleus. They had numerous endoplasmic reticula, mitochondria, and lysosomes (Figure 6A). After 8 weeks of treatment with AFB1, the hepatocyte exhibited signs of vacuolation (Figure 6B) and became largely necrosed, displaying ruptures of the plasma membrane, vacuolation, karyolysis, and the release of cellular contents (Figure 6C). Ito cells had processes that contained lipid droplets and extended between the hepatocytes (Figure 6A,B) or around blood sinusoids (Figure 7B). In the treated group, these cells were overloaded with lipids (Figure 6B and Figure 7B) and exhibited collagen fibers (Figure 6B). Under control conditions, the blood sinusoids exhibited an integral endothelial lining that was perforated by small pores (Figure 7A). However, in the treated group, just a few fenestrae could be detected as most of the pores were disrupted, which had led to the formation of large gaps (Figure 7B). Telocytes were observed around the blood sinusoid (Figure 7A). They have a spindle-cell body, with an elongated euchromatic nucleus and two cytoplasmic processes known as telopodes (Tps).

The space of Disse, i.e., the space located between the hepatocytes and sinusoids, was infiltrated by some cells in the treated group. Kupffer cells were located in hepatic sinusoids (Figure 6B, Figure 7A, and Figure 8A) and projected into the sinusoidal lumen. These cells have irregular surfaces and indented nuclei. They significantly differ in their diameter, density, and shape. In the treated groups, hyperactive Kupffer cells were observed in the space of Disse, which was characterized by large processes and contained lysosomes and phagosomes in addition to phagocytic materials (Figure 8A). Mast cells, plasma cells, and dendritic cells (DCs) had also infiltrated the space of Disse (Figure 8). DCs had an irregular shape with a heterochromatic nucleus and multiple dendrites, such as cytoplasmic processes. These processes came into contact with lymphocytes (Figure 8B). Pit cells with characteristic granules were observed around the blood sinusoid (Figure 7B) and the space of Disse (Figure 8C) in the treated groups. Telocytes were observed around the hepatocyte and blood sinusoids with characteristic cell bodies and cell processes (telopodes). Telocytes demonstrated some morphological changes in the treated groups, including the dissolution of the plasma membrane, which surrounded the cell bodies and contained scant perinuclear cytoplasm. In addition, their cytoplasm showed vacuoles and the dissociation of the telopodes (Figure 8D,E).

The interlobular bile duct, which was lined by pyramidal cells with basally located nuclei, was resting on the basal lamina and was surrounded by a fibrous sheath that increased in thickness in the treated groups (Figure 9).

## 4. Discussion

In the current study, we identified the ultrastructural damage in hepatic parenchymal and nonparenchymal cells, as well as sinusoidal and biliary damage, after exposure to AFB1 in rats. The liver function depends on the interactions between nonparenchymal cells, hepatocytes, and the extracellular matrix they secrete. Thus, hepatocyte damage would not be detected without minimal sinusoidal and perisinusoidal lesions [27]. AFB1 is an extremely hepatotoxic agent that triggers numerous pathological changes in the liver. Moreover, hyperplasia of the bile duct, as well as fibrosis around the portal area, has also been observed with AFB1 exposure [5,28,29,30].

The present study revealed that vacuolar degeneration and necrosis occurred in hepatocytes after oral administration of AFB1 for 4 or 8 weeks. Vacuolar hepatocellular degeneration was significantly high in the 8-week treatment group. This result is consistent with our ultrastructural observations of the hepatocytes, which were largely necrosed and demonstrated rupture of the plasma membrane, vacuolation, karyolysis, and release of cellular contents. Necrosis has been described as an unregulated type of cell death, with various cellular actions that inhibit the swelling of the cell and the rupture of the plasma membrane [31]. Necrosis is a mode of death that occurs due to extreme ATP exhaustion, for example, during toxic injury and oxidative stress with ROS formation [32]. It results in changes to cell membrane integrity that lead to ion pump damage, which is the initial process in vacuolar degeneration and cell swelling [33].

Substantial megalocytosis and binucleation of hepatocytes were observed in AFB1-treated groups; the number of megalocytes and binucleated cells was significantly higher in the 8-week treatment AFB1 group compared with the other groups. These results are in agreement with those of Kalengayi and Desmet [34], who reported that AFB1 induces tumor formation, in which cells demonstrate abundant eosinophilic cytoplasm, enlarged nuclei with prominent nucleoli, and abnormal mitosis. The regeneration of AFB1-damaged hepatocytes by natural proliferation is narrowed, particularly during prolonged aflatoxicosis [35,36]. Megalocytosis occurs as a consequence of the nuclear and cellular enlargement of cells, which exhibit dynamic DNA and protein biosynthesis [37].

It has previously been reported that AFB1 toxicity induces DNA damage [14,38]. In our study, mitotic abnormalities were documented in cells. In a previous study, the proportion of total abnormalities was relatively high and increased as the duration of AFB1 exposure was extended [39].

In the present study, AFB1 treatment caused abnormalities in the sinusoidal endothelium and in the sinusoidal and perisinusoidal cells. According to our ultrastructural observations, most of the endothelial fenestrae were disrupted, and large gaps were formed. This damage occurs in the endothelial lining, which leads to the disruption of the endothelial barrier; it has been previously reported as a consequence of pathological conditions, such as exposure to Kavian [40]. The liver endothelial filter is considered to be a critical factor in the distribution of chylomicron fragments, which in turn may lead to a fatty liver [41]. In the present study, we reported perisinusoidal fibrosis with AFB1 treatment; this has been previously observed with numerous pathological conditions, including alcoholic fibrosis and hepatocellular carcinoma [42].

Within and surrounding the blood sinusoids, we observed cells other than hepatocytes, e.g., Kupffer cells, fat-storing cells (Ito cells), and pit cells. Each cell type has a characteristic fine structure [41]. Kupffer cells are resident macrophages; they are located within the blood sinusoids and connect with the endothelium through their cytoplasmic processes [43]. In the current study, Kupffer cells infiltrated the space of Disse in AFB1-treated groups, which may have been caused by the destruction that occurred in the sinusoidal barrier. In other specific pathological conditions, Kupffer cells partially or completely infiltrate the space of Disse [42]. Conversely, the activated Kupffer cells were observed to contain many lysosomes and phagosomes. It has been well established that Kupffer cells act as both defenders against, and mediators of, hepatic damage. For instance, the dysfunction or exhaustion of Kupffer cells protects the liver against injury that can be caused by the alkylating agent melphalan [44]. In addition, the activation of Kupffer cells by toxic agents influences the release of certain inflammatory mediators, growth factors, and ROS. Such an activation helps in controlling the acute and chronic liver responses involved in hepatic cancer [45]. During cellular degeneration and necrosis, we found that Kupffer cells proliferated after AFB1 treatment, with proliferation increasing as the duration of toxicity increased. This observation is in agreement with a previous study [46]. The activated Kupffer cells in turn activate fat-storing cells to release their product, which will have already occurred during tissue damage [40]. Fat-storing cells, i.e., perisinusoidal cells or Ito cells, contain fat droplets; under pathological conditions, they become overloaded with these droplets [43]. This finding is contrary to our ultrastructural results in AFB1-treated groups. Nevertheless, Ito cells with overloaded lipids have been observed in patients with hypervitaminosis A and hepatocellular carcinoma [42]. In addition, the space of Disse in our AFB1-treated groups was infiltrated by immune cells such as pit cells, DCs, mast cells, and plasma cells. Pit cells are natural defense cells that show the morphology of granular lymphocytes containing granules; they have cytotoxic activity against immigrating tumor cells [47]. DCs are antigen-presenting cells that are a factor in the induction and regulation of immune responses [48]. The presence of mast and plasma cells was previously reported in alcoholic hepatitis and chronic hepatitis [42]. Taken together with our findings, these results indicate that aflatoxin hepatotoxicity may be immune-mediated.

Telocytes are an interstitial cell type found in various organs; they are involved in several tissue functions in addition to playing pathophysiological roles in several disorders [49]. Hepatic telocytes play a role in the function of adjacent hepatic stellate cells. Thus, the loss of telocyte function leads to stellate cell dysregulation [50]. In our study, telocytes were identified in the space of Disse in the liver, and exhibited morphological changes in AFB1-treated rats. A recent study demonstrated the role of telocytes in hepatic fibrosis; the disappearance of telocytes may influence liver hemostasis and its regeneration [51].

Different degrees of periportal fibrosis and bile duct hyperplasia were observed in AFB1-treated groups [52]. This reaction is assumed to restore damaged hepatocytes in the vicinity during liver injury [53]. Thus, these changes can be attributed to AFB1-induced hepatic injury [34,54].

Overall, histopathologic hepatocellular injury was severe in AFB1-treated rats administered 250 µg/kg body weight/day for 8 weeks. The liver is a complex organ; its function is regulated by complex interactions between the hepatocytes and nonparenchymal cells. When hepatocytes are damaged, the other cells in the liver will be affected; they may even proliferate, which leads to the formation of an excessive amount of connective tissue. In conclusion, AFB1 interferes with the homeostasis and cellular milieu of the liver, leading to severe liver damage.

## Data Availability

The data that support the findings of this study are available on request from the corresponding author. The data are not publicly available due to privacy or ethical restrictions.
